# Explicit equilibrium modeling of transcription-factor binding and gene regulation

**DOI:** 10.1186/gb-2005-6-10-r87

**Published:** 2005-09-30

**Authors:** Joshua A Granek, Neil D Clarke

**Affiliations:** 1 Department of Biophysics and Biophysical Chemistry, Johns Hopkins University School of Medicine, North Wolfe Street, Baltimore, MD 21205, USA; 2 National Evolutionary Synthesis Center, Broad Street, Durham, NC 27705, USA; 3 Genome Institute of Singapore, Biopolis Street, Singapore 138672, Republic of Singapore

## Abstract

We have developed a computational model that predicts the probability of transcription factor binding to any site in the genome. GOMER (generalizable occupancy model of expression regulation) calculates binding probabilities on the basis of position weight matrices, and incorporates the effects of cooperativity and competition by explicit calculation of coupled binding equilibria. GOMER can be used to test hypotheses regarding gene regulation that build upon this physically principled prediction of protein-DNA binding.

## Abstract 2

A computational model, GOMER, is presented that predicts transcription-factor binding and incorporates effects of cooperativity and competition.

## Background

Transcription is regulated by the binding of proteins to specific DNA sequences. Until recently, binding and regulation could only be studied at the level of individual genes, but they can now be studied as a complex system due to the availability of genome-wide data on expression and transcription factor binding. Computational models are needed, however, to evaluate co-regulated genes and the sequence motifs associated with them.

A general strategy for testing the relevance of a DNA binding motif to gene regulation is to quantify the association of the motif with co-regulated genes. This can be done by comparing the regulatory sequences of co-regulated genes with the regulatory sequences of all other genes [1-4]. One simple test is to score for the occurrence of a consensus site within a prescribed distance 5' to the start of transcription. If the fraction of regulated genes with a consensus site is significantly larger than the fraction of unregulated genes, as it often is, then the test has some predictive power [1,5-7]. As with all statistical tests, there is a model implicit in this test: in this case, the implicit model is that gene regulation is mediated by a single consensus binding site.

There are problems with such a simple model. First, the use of consensus binding sites, even if degenerate, underestimates the importance of motifs that resemble the consensus but do not match it [8]. At the same time, degenerate consensus sites fail to distinguish among motifs that match the consensus even if the motifs that match differ in affinity. Second, regulated genes often contain more than one binding site for a given factor, so scoring based on a single site (or any other threshold number of sites) is arbitrary. Third, the binding of a factor is typically affected by cooperative and competitive interactions with other proteins, so binding sites for those other proteins may need to be considered. Fourth, gene expression can be affected by the location, orientation and spacing of bound transcription factors. Therefore, to be realistic, a model for gene regulation should use to full advantage an accurate representation of binding specificity, integrate over multiple binding sites of different strength, account for cooperative and competitive interactions, and be flexible enough to model the variable effects that binding can have on gene expression.

We previously described an algorithm for predicting the probability that a transcription factor binds within a promoter region [3]. The algorithm predicts the relative affinity of binding sites using a position weight matrix (PWM) in which the elements of the PWM represent contributions to the free energy of binding for all possible bases at each position in a binding site [9]. The algorithm then integrates over the affinities of all possible binding sites within a region of interest by calculating the probability that at least one site is bound at a given assumed protein concentration. Using a PWM defined by extensive binding equilibrium measurements of yeast Leu3p, we showed that this method was able to predict the set of known target genes for Leu3p better than could be achieved by simple enumeration of discrete binding sites [3].

Building on those results, we report here a very general physically principled model for transcription factor localization based on protein-DNA and protein-protein binding equilibria. The model, which we have named GOMER (generalizable occupancy modeling of expression regulation), uses PWMs to predict explicitly the relative affinity of binding sites, taking into account the effect of cooperative and competitive interactions. Based on the binding predictions, GOMER predicts gene regulation by weighting binding sites according to their location and orientation. The weights are calculated from functions specified or defined by the user. These functions and their parameters allow the user to test alternative hypotheses concerning the control of co-regulated genes.

Here we describe GOMER and give examples of its application. We use the program to analyze the effect of cooperativity between forkhead proteins and the transcription factor Mcm1p in controlling the expression of a set of cell-cycle regulated genes in yeast [7,10]. Although *in vitro *experiments show that direct interactions between these factors occur over very short distances [11], we find evidence that cooperative interactions can extend over a distance of 100 base pairs (bp) or more. We also use the model to investigate the role of competition between two transcription factors, Ndt80p and Sum1p, in distinguishing between mitotic and meiotic programs of gene expression [12]. Competition between these proteins better explains a set of genes that is regulated by both transcription factors than does simple non-competitive binding. Finally, we evaluate the correlation between predicted and observed binding of Rap1p in a chromatin immunoprecipitation microarray (ChIP-array) experiment [13]. We show that the correlation between predicted and observed binding can be dramatically improved by a model that accounts for hybridization to a spot on the array (an array feature) that is due to binding to sites outside the sequence of the array feature itself. The GOMER program is freely available.

## Results

### Realistic modeling of promoter regions using binding site weight functions

**Figure 1:**
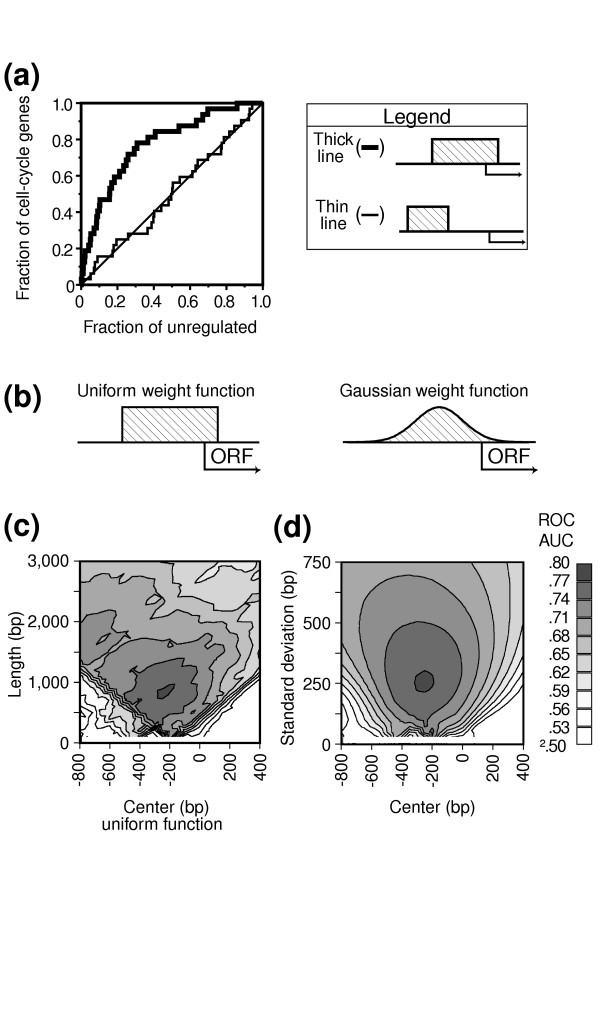
Alternative definitions of the regulatory region and their effect on the prediction of gene regulation. **(a) **Receiver operating characteristic (ROC) curves showing how *CLB2 *cluster genes rank compared to all other genes using the forkhead probability matrix and two different definitions of the regulatory region. ROC curves plot the fraction of true positives that meet a threshold value (here, a given GOMER score) against the fraction of false positives that meet that same threshold. The thick line plots a ROC curve for a regulatory region defined as the sequence between 650 base pairs (bp) 5' to the ORF and 150 bp 3' to the start of the ORF; the thin line plots a ROC curve for a regulatory region defined as the sequence between 1,000 bp and 500 bp 5' to the ORF. The latter definition of the regulatory region has no predictive value as reflected in the nearly diagonal ROC curve (area under the ROC curve (ROC AUC) of approximately 0.5). **(b) **Schematics of a conventional uniform weight function and a Gaussian weight function. **(c) **Comparison of the uniform weight function and **(d) **the Gaussian weight function for several hundred combinations of parameter values. The contoured areas are shaded according to ROC AUC value as indicated on the scale. To facilitate comparison, the regulatory regions defined by the uniform weight function are plotted in terms of the center of the region, analogous to the center of the Gaussian distribution. Center values are expressed as distance from the open reading frame (ORF); negative values are 5' to the ORF start. For the Gaussian function, weights below 1/1,000^th ^the maximum value are rounded down to 0.

A group of yeast genes named the *CLB2 *cluster is normally expressed in a cell-cycle dependent fashion but loses its cell-cycle dependence in a *fkh1Δfkh2Δ *mutant lacking forkhead transcription factors [7,10]. To assess the association of forkhead binding sites with forkhead-dependent cell-cycle regulation, we used GOMER to score all putative regulatory sequences using a forkhead PWM that was defined by binding data for Fkh1p. The data for Fkh2p is not as complete but the proteins have similar specificity [11]. The ranks of *CLB2 *cluster genes, based on the GOMER occupancy score, were compared to all other genes in the genome using a receiver operating characteristic (ROC) curve (Figure 1a) [14]. In this context, a ROC curve is a series of connected points, each of which shows the fraction of regulated genes that meet or exceed a given GOMER occupancy score versus the fraction of unregulated genes that meet or exceed the same score; these values are plotted for all observed occupancy scores. The ROC curve can also be thought of as a graphical representation of how the ranks of regulated genes are skewed with respect to the ranks of other genes in the genome when genes are ranked by their GOMER occupancy score. One way to quantify this skewing of ranks is by calculating the area under the ROC curve (ROC AUC). We have previously discussed the merits of the ROC AUC value as a criterion for evaluating models of gene regulation, and the metric is used here extensively [15].

In GOMER, regulatory regions are defined by user specified functions that assign a weight to each binding site based on its location. For example, it is common practice to assume that yeast regulatory regions consist of the 600 bp 5' to the start of translation [1,5,16]. To model this regulatory region in GOMER we used a function that simply assigns a weight of 1 to all sites that lie within the region and 0 to all sites outside. The region itself is defined by parameters to the function that specify the endpoints of the region with respect to the 5' end of an open reading frame (ORF). Figures 1a and 1c show the effect of varying the parameters for this simple model (the beginning and end points of the regulatory region).

While the conventional 600 bp definition of the regulatory region works well (ROC AUC = 0.75), alternative parameters explain the *CLB2 *cluster genes somewhat better. The choice of parameters that works best defines a regulatory region extending from 650 bp 5' to the ORF to 150 bp inside the ORF (ROC AUC = 0.78). Exclusion of the 150 bp inside the ORF makes the model perform somewhat less well (ROC AUC = 0.75), which means that sites within the first 150 bp contribute to our ability to distinguish true forkhead regulated genes from other genes that happen to have forkhead binding sites. Thus, there may be weak but biologically relevant binding sites within the coding region of some forkhead-regulated genes.

Real regulatory regions rarely have strict boundaries like the 600 bp definition used by convention in yeast, and sites within these regions can differ substantially in their functional importance. One advantage of the GOMER approach is that it allows users to evaluate more realistic models of gene regulation by defining their own regulatory-region weight functions. Figure 1b illustrates, as an example, a Gaussian weight function, and Figure 1d shows the results of using this function with various parameters. The Gaussian weight function models a regulatory mechanism in which there is an optimal position for a bound protein to affect gene expression. The effect of a bound protein decreases with distance from this optimal position. Unlike the uniform weight function, there is no sudden and substantial drop in weights (though weights below a user-specified threshold are rounded down to zero in the interests of computational efficiency).

Figures 1c and 1d compare the effectiveness of the uniform and Gaussian functions over an equivalent range of parameters. The two functions achieve similar ROC AUC values using their optimal parameters, but the uniform weight function is much more sensitive to the choice of parameter values than is the Gaussian function. This is evident from the irregular contours in Figure 1c, which are a consequence of the hard cutoffs imposed by the uniform weight function. Thus, GOMER's flexible definition of gene regulatory regions allows for regulatory models that are both more realistic and more robust.

### Homotypic and heterotypic cooperativity in the regulation of cell-cycle genes by forkhead transcription factors

The forkhead PWM is able to distinguish *CLB2 *cluster genes reasonably well using either the uniform-weight definition of the regulatory region or the Gaussian-weight definition. However, neither model is exceptionally good: both have ROC AUC values of approximately 0.78. *In vitro *experiments suggest that Fkh2p binds cooperatively to DNA with itself and with the transcription factor Mcm1p [11,17]. The lack of cooperative interactions in the models might explain their suboptimal performance.

**Figure 2:**
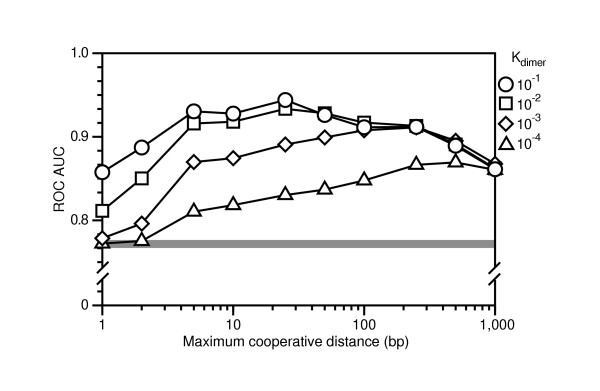
Modeling Fkh2p-Mcm1p cooperativity improves the ability to identify cell-cycle genes. Scores for the area under the receiver operating characteristic curve (ROC AUC) are plotted as a function of the maximum distance over which cooperative interactions between Fkh2p and Mcm1p are allowed to occur. Different symbols correspond to different assumed values for K_dimer_, a parameter that specifies the strength of cooperative interactions (10^-1 ^(circles), 10^-2 ^(squares), 10^-3 ^(diamonds) and 10^-4 ^(triangles)). The horizontal gray line indicates the ROC AUC value in the absence of cooperative interactions with Mcm1p. All calculations were performed using the optimal regulatory region definition previously determined for non-cooperative binding (Gaussian weight function with mean = 250 base pairs (bp) and SD = 250 bp).

To see whether performance of the model could be improved by including homotypic (Fkh2p-Fkh2p) or heterotypic (Fkh2p-Mcm1) interactions, we used GOMER to model these interactions, varying the strength of the dimerization constant and the allowed distance between cooperatively interacting binding sites (Figure 2). The inclusion of homotypic cooperativity had little effect on our ability to explain regulation of the forkhead-regulated genes (not shown). The inclusion of heterotypic interactions with Mcm1p, however, dramatically improves the quality of the model. For parameter values that model a strongly cooperative interaction, the ROC AUC achieves its highest value when the maximum allowed distance between Fkh2 and Mcm1 binding sites is 25 bp. If we assume the interaction is weaker, the maximum ROC AUC value is not quite as high but it increases steadily to a maximum distance between binding sites of 500 bp. This result was unexpected because *in vitro *binding experiments had suggested preferences for close and precise spacing in the cooperative interaction of Fkh2p and Mcm1p [17]. One possibility is that Fkh2p and Mcm1 bind cooperatively by two different mechanisms: through direct interaction over short distances; and indirectly over longer distances. One plausible mechanism for indirect, long-range cooperative interaction is through mutual competition with nucleosome binding [18]. Thus, the computational analysis supports the idea that cooperativity is an important feature of the regulation of these genes, and suggests that cooperative effects may occur over a longer range than had been anticipated.

The model for cooperativity used in this analysis is extremely simple: all sites within a given distance are considered to be equally capable of interacting cooperatively. However, GOMER's 'plug-in' weight functions make it easy to explore more elaborate models for cooperativity (see Materials and methods).

### Competitive interactions between Sum1 and Ndt80

Competition among transcription factors is a potentially important mechanism for controlling complex biological responses. We have incorporated a realistic model for competitive interactions into GOMER (see Materials and methods) and have used this model to study the interaction of yeast transcription factors Ndt80p and Sum1p. Ndt80p is an activator of genes expressed during the middle stage of sporulation [5,19]. Sum1p represses genes during mitotic growth and the early stage of sporulation [12,20]. A number of the genes induced by Ndt80p during middle sporulation are targets of repression by Sum1p. Ndt80p and Sum1p have overlapping binding specificities, which suggests that competition between these transcription factors may be important for regulation. Competition for binding has been demonstrated *in vitro *by gel-shift assays and *in vivo *using reporter constructs [12].

**Figure 3:**
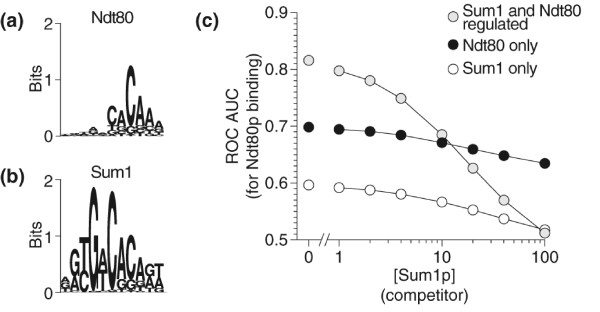
Effect of competition by Sum1p on predicted binding by Ndt80p. Sequence logos [37] for **(a) **Ndt80p and **(b) **Sum1p binding specificity. **(c) **Values for the area under the receiver operating characteristic curve (ROC AUC) quantify how well predicted Ndt80 binding distinguishes regulated genes from non-regulated genes. The regulated gene sets are the genes controlled by Ndt80 only (black), Sum1 only (white), or both (gray). For all comparisons, the set of non-regulated genes consists of genes not regulated by Ndt80 or by Sum1. Sum1p concentration is expressed as a ratio to the optimal predicted K_d _value for Sum1p binding; Ndt80p concentration is set equal to the optimal predicted K_d _value for Ndt80p binding. The regulatory region was defined by the uniform weight function over the sequence between 600 base pairs 5' to the open reading frame and the start of translation.

We first calculated the GOMER occupancy scores for all yeast genes using either a Sum1p PWM alone or an Ndt80p PWM alone. As expected, the Sum1p PWM does a good job of identifying genes that are regulated by Sum1p (including those that are also regulated by Ndt80p), but it does a poor job of identifying genes that are regulated by Ndt80p only (not shown). Conversely, the Ndt80p PWM does a poor job of identifying genes that are regulated only by Sum1p, and a reasonably good job of identifying Ndt80p regulated genes (including those that are also regulated by Sum1p). In fact, genes that are regulated by Sum1p in addition to Ndt80p are better explained by Ndt80p binding sites than are the genes regulated by Ndt80p alone (Figure 3).

If competition between Sum1p and Ndt80p were relevant to the regulation of a particular gene, we would expect the regulatory sequence for that gene to be sensitive to the concentrations of the two transcription factors. To test this, we fixed the concentration of Ndt80p in the model and explored the effect of increasing concentrations of competing Sum1p. Importantly, the genes that are regulated by both proteins, and therefore are the best candidates for being affected by competition between the proteins, show the greatest sensitivity to competition by Sum1p (Figure 3). At higher Sum1p concentrations there is substantially less specific binding by Ndt80p to these genes, as reflected in lower ROC AUC values for this set of genes when scored for Ndt80p occupancy. Substantially smaller effects of Sum1p concentration are seen for the genes that are regulated by Ndt80p alone, consistent with the observation that Sum1p does not regulate these genes. A similar conclusion was recently reported independently by Wang *et al*. [21]. These results suggest that binding site variants that are found in genes regulated by both Ndt80p and Sum1p have been tuned by evolution to be sensitive to the relative concentration of the two proteins. Binding sites in genes regulated by only one of the transcription factors tend to more closely match the specificity of that particular transcription factor and are, therefore, less sensitive to the effects of the competing factor.

### Improved correlation between predicted and observed binding in ChIP-array experiments

**Figure 4:**
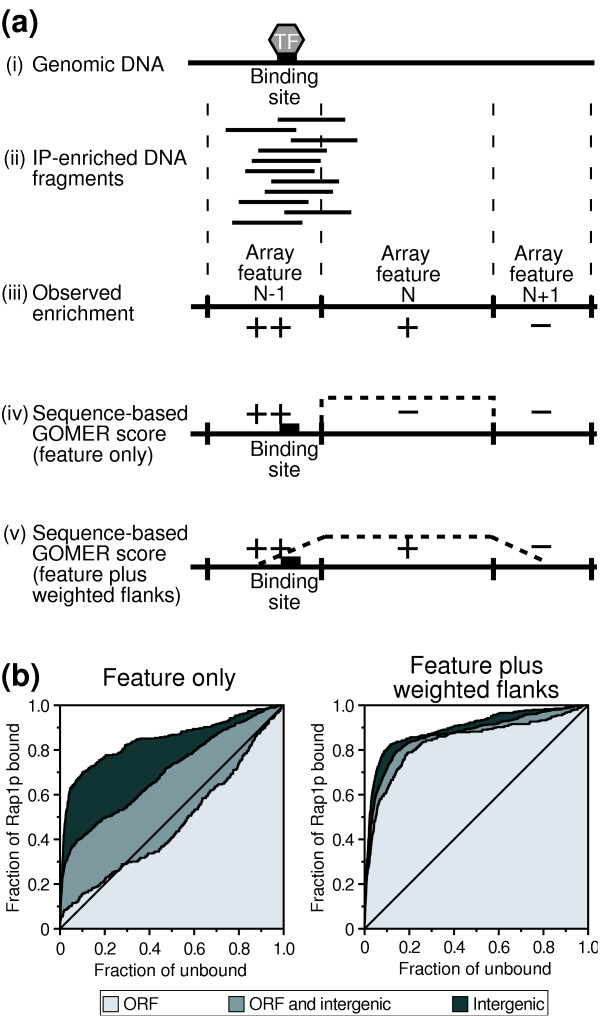
Application of GOMER to chromatin immunoprecipitation microarray experiments. **(a) **The contribution to array feature enrichment by binding sites outside the sequence of the array feature. (i) A single protein bound to a single high-affinity site (ii) yields a population of enriched DNA molecules averaging approximately 500 base pairs in length. (iii) Hybridization of the enriched sequences to a DNA microarray results in a signal for those array features that overlap the enriched DNA sequences (N-1 and N). (iv) If the sequence of the array features alone is used to predict binding, enrichment of feature N cannot be accurately predicted. (v) Enrichment can be predicted if flanking sequences are included in the calculation. Binding sites outside the array feature sequence are down-weighted as a function of distance from the array feature boundary. **(b) **Receiver operating characteristic (ROC) curves for Rap1p enriched versus unenriched features, with features ranked by GOMER scores. GOMER scores were calculated using only the features themselves (left) or the features plus weighted flanking sequences (right). ROC curves for different subsets are indicated by shading under the curve: open reading frame (ORF) features only (light gray); both intergenic and ORF features (medium gray); intergenic features only (dark gray).

The GOMER model was designed to provide flexibility in modeling gene regulation, but it can also be used to model the genome-wide binding of transcription factors. As an example, we have used it to analyze the *in vivo *binding of Rap1p as determined by whole-genome ChIP-array [13]. Using a Rap1p PWM we determined GOMER scores for the genomic sequences represented on the array and used ROC curves to evaluate the association of predicted binding with Rap1p immunoprecipitation (Figure 4). On the whole, enrichment of genomic sequences is reasonably well explained by the model for Rap1p binding (ROC AUC = 0.70). However, this is an average value: array features (spots on the array) that correspond to intergenic sequences score exceptionally well (ROC AUC = 0.84), but features that correspond to coding sequence score no better than random (ROC AUC = 0.47).

This difference is largely due to the naïve model we used initially to score sequence features on the array. This model considers only the sequence of the array feature itself (Figure 4a). Because bound DNA is sheared to a size of several hundred base pairs in the ChIP procedure, some of the molecules that are immunoprecipitated due to binding to a site within one array feature overlap the sequence of a neighboring feature, as previously pointed out by Lieb *et al. *[13]. We can model this effect in GOMER using a weight function that allows sites outside the feature to contribute in proportion to the fraction of immunoprecipitated molecules we expect to hybridize. Doing so dramatically improves our ability to explain the Rap1p ChIP data, especially for ORF features (Figure 4b, right). This suggests that much of the experimental enrichment of ORF features is actually due to binding sites that are in sequences flanking the ORFs (that is, in intergenic regions).

## Discussion

The GOMER scoring function uses PWM scores to estimate relative free energies of binding to potential sites. How well this works depends on how well the PWM represents the contributions of each base to the free energy of binding. These free energy contributions can be directly estimated from thermodynamic binding data, but more commonly they are inferred from the base frequencies in known binding sites. This is possible because there is a connection between the information content in a set of sequences and the thermodynamics and specificity of binding (see Materials and methods for a fuller discussion) [9]. A variation on this idea treats the PWM as a predictor of affinity and then uses protein concentration as a variable to maximize the likelihood of observing the set of known sequences [22]. Regardless of how the PWM is defined, GOMER itself can be used to compare the predictive value of different PWMs by assessing their ability to explain experimental binding or expression data. For example, we have shown that PWMs defined either by direct measurement of binding affinities or by computational motif discovery are equally good at explaining an independent ChIP experiment. [23]

A key attribute of GOMER is its flexibility. GOMER uses weight functions, specified by the user, to create position-dependent models that define the size and shape of regulatory regions and describe the nature of cooperative and competitive interactions (see Materials and methods). These functions can be as complex as the user desires, although care should be taken not to use more parameters than is justified by the data. The power of this approach for modeling gene regulation will become more valuable as more data become available.

One parameter used by GOMER is the free concentration of transcription factors, which is needed for calculating binding site occupancies based on predicted affinities (see Materials and methods). When a single, non-cooperative factor is analyzed, concentration has only a marginal effect on the ROC curve. This is because only the ranks of the genes are relevant to the curve, not the absolute occupancy of the gene by the transcription factor. (There can be a modest effect of concentration in this case because the occupancy score for a gene with a single high-affinity site changes with concentration somewhat differently than does the occupancy score of a gene with several lower-affinity sites [3].) Varying the concentration can, however, have a much more substantial effect when cooperative and competitive interactions are included in the model (Figures 2 and 3). Because cooperative and competitive interactions are common in gene regulation, the explicit consideration of concentration is likely to be necessary for a complete understanding of gene regulation.

GOMER is a physically principled method because of the way it uses PWMs to estimate binding affinities but also because its weight functions and parameters can be understood in terms of specific physical and biological models. This distinguishes GOMER from machine learning methods that search for rules describing gene regulation without the assumption of an underlying physical model [4]. GOMER also differs in philosophy from purely empirical algorithms. For example, rules for defining clusters of binding sites have been developed that help distinguish regulated genes from other genes that have a comparable number of binding sites [24-26]. GOMER, on the other hand, can distinguish genes with clustered binding sites from genes whose sites are dispersed by modeling cooperative binding interactions. These cooperative interactions are likely to be the reason why sites are clustered in the first place.

We showed previously that gene scoring functions that are based on enumeration of binding sites are typically poorer predictors of gene regulation than is the simple GOMER occupancy score, which integrates over binding sites of differing predicted affinities [3]. We expect, of course, that any motif searching algorithm that uses PWMs in a related way, and which ranks genes based on the scores for all sites, would perform similarly. To verify this, we ran the motif searching program PATSER using the FKH1 and NDT80 PWM, and obtained scores for the top five sites upstream of every gene [27]. PATSER does not provide an integrated binding site score for each gene, so we ranked genes according to their highest scoring site. In the event of ties, the second highest scoring site was used as a tie breaker, then the third highest scoring site, and so on. For the largest gene set analyzed, the genes regulated by NDT80 only, the ROC AUC values for the simple GOMER function and the PATSER-based ranking algorithm are nearly identical (between 0.70 and 0.71). For two smaller gene sets, the simple GOMER function performed better than the PATSER-based algorithm in one case (0.78 versus 0.72 for the *CLB2 *cluster genes) and less well in the other (0.80 versus 0.89 for the genes regulated by both NDT80 and SUM1).

The purpose of this paper is to demonstrate that a substantial improvement in these scores can be obtained using GOMER's cooperative and competitive modeling functions. GOMER is unique thus far in its ability to model cooperative and competitive interactions, so we are not able to compare these important features of GOMER to other algorithms. We hope the availability of GOMER and the data sets used in this paper will permit others in the field to test GOMER against new algorithms as these new algorithms are developed.

## Conclusion

Computational models of gene regulation are far from perfect because gene regulation is a complex phenomenon. It is because of this complexity, though, that it is important to develop realistic, quantitative models like GOMER. By assessing how well (or poorly) we can predict the effect of mutations or environmental signals, we can better identify deficiencies in our understanding of gene regulation and allow the development of new additions to the model. GOMER can be applied to other organisms besides yeast, and indeed we have begun using it to study developmentally important transcription factors in *Caenorhabditis elegans*. GOMER can also be used to study sequence signals that regulate transcription termination [28], and it could be adapted to study any regulatory mechanism that involves sequence specific binding, not just transcription. In the future, we anticipate incorporating experimental data on the distribution of nucleosomes and nucleosome modifications, and we will begin to address differences in the kinetics of binding in addition to differences in affinity.

## Materials and methods

### Representation of binding specificity and the prediction of binding affinity

The elements of a PWM are base-specific scores for each position of a potential binding site. The values of the PWM can be defined by direct measurement of binding affinities [3,12], but more often they are estimated from the frequency of occurrence of each base at each position in a set of presumptive binding sites. These sites are determined experimentally, for example by binding site selection [29] or by computational analysis of co-regulated genes [2,30,31], or by a combination of selection and computational analysis [23]. Typically, a PWM element [**b**,**j**], is derived from the ratio f_b,j_/p_b _where f_b,j _is the observed frequency of base **b **at position **j**, and p_b _is the prior probability of base **b **(usually the frequency of **b **in the genome). The ratio f_b,j_/p_b _can be thought of as an equilibrium constant between the protein binding to sites that contain base **b **at position **j **and the protein binding to a mixture of sites containing each of the four bases at position **j**, with the frequency of the bases the same as that found in the genome [9]. It follows that if PWM elements are calculated as RTln(f_b,j_/p_b_) (where R is the gas constant and T is the temperature), the value of element [**b**,**j**] can be interpreted as the contribution to the relative free energy of binding to a base **b **at position **j **in a particular sequence. In practice, GOMER generates the PWM internally from data supplied by the user: a probability matrix (PM) file (which contains the position-specific base frequencies), and the expected base frequencies (calculated from the sequences). A temperature of 300 K is used in calculating the PWM from the PM.

Sequence windows (potential binding sites) are scored by summing the appropriate base-specific values for each position in the window, as defined by the PWM. The score for a site is computed as the sum of position scores, based on the assumption that each base makes an independent contribution to the free energy of binding to the site. This assumption is a good approximation in most cases [32]. The PWM score for a window can be interpreted, therefore, as a relative free energy of binding and from that value an equilibrium binding constant (K_d _= e^-ΔG/RT^) can be calculated. A default temperature of 300 K is used to calculate the equilibrium constant from the PWM; however, the temperature parameter can be varied, changing the relative affinity for favored bases over disfavored.

### Probability of protein occupancy for regulatory sequences

Once an equilibrium constant has been calculated for a sequence window, **i**, the probability of binding to that site, P_i_, can be calculated from the standard equation for a simple binding isotherm:



where K_d,X,i _is the predicted equilibrium dissociation constant for X binding to window i and [X] is the free concentration of X. Although [X] represents a real physical quantity, it is exceedingly difficult to determine its *in vivo *value experimentally [33], so for most purposes [X] is an adjustable parameter. By default, [X] is set equal to the K_d,X _for the optimal binding site, resulting in an occupancy score of 0.5 for optimal sites.

The probability of binding is calculated for all sequence windows within a regulatory sequence. GOMER then integrates over all sequence windows by calculating the probability, P_occ_, that the protein is bound to at least one site within the regulatory sequence based on the probability of binding to each site,  P_i_.



The probability of not being bound at site i, 1 - P_i_, is



where K_a,X,i_, is an equilibrium association constant and is the reciprocal of K_d,X,i_.

Therefore:



(We used K_d_, the equilibrium dissociation constant, at the beginning of the derivation because its use in the standard binding isotherm equation is familiar to biochemists, but we switch here to K_a_, the equilibrium association constant, because the final form of the GOMER scoring function is visually less complicated using this substitution).

### Regulatory regions are defined in GOMER by user-specified weight functions

Generally, we want to use GOMER to predict the probability of a gene being regulated rather than just the probability that a transcription factor binds in its vicinity. To determine this functional probability, we need to weight binding sites by their expected relevance to regulation. In GOMER, equilibrium constants are modified by weights calculated from user-specified functions. These functions weight sites based on their location and/or orientation with respect to genome features (for example, the start of transcription). Thus, we define a GOMER score, S, which is similar to P_occ _but which incorporates functional weights.



where K_a,eff,X,i _= κ_i_K_a,X,i _and κ_i _is the weight for site **i **based on the user-specified function.

### Cooperative interactions

The cooperative binding of proteins X and Y to DNA can be separated thermodynamically into the formation of an XY dimer and the binding of that dimer to DNA; this is thermodynamically equivalent to protein X binding to its site with higher affinity in the presence of pre-bound Y. This leads to a conceptually simple means for incorporating cooperative interactions into the GOMER model: the probability that a given site **i **is occupied by X depends not only on the probability that it is occupied by monomeric X but also on the probability that it is occupied by XY. Calculating the probability of occupancy by the XY dimer requires us to take into account all possible pairs of binding sites that consist of a site **i **to which X binds and a second site, **j**, to which Y binds. These site pairs need not be contiguous. Extending the expression derived above for monomer binding, the expression for calculating the GOMER score, accounting for cooperative interactions, is:



K_a,eff,XY,i,j _is the equilibrium constant for an XY dimer binding to a site that consists of windows **i **and **j**; it is analogous to K_a,eff,X,i_, the equilibrium constant for monomeric X binding to site **i **in that it is the product of an intrinsic binding affinity, K_a,XY,i,j_, and a weight, κ_C,i,j_, which is assigned by a user-specified weight function κ_C_. GOMER assumes that the intrinsic binding affinity of the dimer, K_a,XY,i,j_, is the product of the binding constants of the two proteins, X and Y, for their respective sites, **i **and **j**. Thus:

K_a,eff,XY,i,j _= κ_C,i,j_·K_a,X,Y,i,j _= κ_C,i,j_·(K_a,eff,X,i_·K_a,Y,j_)

where the affinity of protein Y for its site j (K_a,Y,j_) is calculated from a PM in the same way as we have described for protein X. There is no need to apply a functional weight to Y binding because the only role for Y in the model is modification of X binding, rather than a direct role in modulating expression.

The weight function, κ_C_, will typically define weights depending on the spacing and orientation of site **j **with respect to site **i**. For example, if two sites must be adjacent for cooperative binding to occur, then a simple weight function can be used that assigns a weight of 1 for adjacent sites and a weight of 0 to all other sites. The concentration of the dimer, [XY], is the product of [X], [Y], and the dimerization constant, K_a,dimer _([XY] = [X][Y]K_a,dimer_). By default, [X] and [Y] are set equal to the K_d _for their respective optimal sites, and K_a,dimer _is equal to the K_a _for binding of monomeric Y to its optimal site. All these values are parameters in the model. The strength of the cooperative interaction can be adjusted by varying the affinity between X and Y (K_a,dimer_).

There is no limit to how many transcription factors can bind cooperatively with protein X. The product is therefore taken over all cooperative factors, Y. Homotypic cooperativity is simply a special case, where the same transcription factor matrix is supplied for both X and Y:



### Competition

For a single competitor protein, Q, binding in direct competition to the same sites as protein X, the higher the concentration of Q or the stronger its affinity for window **i**, the lower the probability that X will be bound to that window. Formally:



where K_a,Q,i _is the predicted binding constant for Q at site **i **based on the PM for protein Q. More generally:



where



The competition term, C_i_, incorporates all potential competitors binding at any window, **k**, that affects binding of protein X to site **i**. κ_Q,i,k _is a weight defined by a user-specified function that determines the effect of protein Q binding at site **k **on the binding of protein X at site **i**. For a simple competition weight function, the weight might be a binary function of the distance between sites **i **and **k**, such that for sites closer than a distance threshold, the weight is 1 (binding of Q completely occludes binding of X) and for sites further than the threshold distance the weight is 0 (no competition). This function models simple steric exclusion, but more complex functions of the distance and orientation between sites can be used to model more complex interactions.

### The complete GOMER model

Adding the effect of competition to the scoring function derived above for cooperative interactions, we obtain the complete model for *in vivo *binding and gene regulation, as implemented by the GOMER program.



GOMER reports the GOMER score, S, for all regulatory sequences that are of interest. These can be specified in several ways: by reference to a gene annotation file (for example, the 1,000 bases 5' to the start of an ORF or a snRNA gene); using a list of genome sequence coordinate pairs (see the analysis of ChIP-array data below); or providing FASTA-formatted sequence files. In addition to the scores for each sequence of interest, GOMER also reports statistical measures that quantify the ability of a model to distinguish sequences that have been classified as regulated from those that are not. Here, we restrict our discussion to the ROC AUC [14]. A fuller discussion of evaluation metrics is available elsewhere [15].

### Genome sequence, regulated gene sets and probability matrices

All analyses were performed using *Saccharomyces cerevisiae *genome sequence and genome annotation files obtained from the *Saccharomyces *Genome Database [34] on January 29, 2004. PMs used in this work and lists of regulated genes are available as Additional data files 2, 3, 4, 5, 6. For analysis of ChIP-array data, the genome sequence coordinates that define each microarray spot were determined from the sequences of the PCR primers used to make the array (see supplementary methods in Additional data file 1 for details).

### Program implementation

The GOMER program was written in Python [35]. Weight functions are Python modules with a defined programming interface so users can create novel functions to fit their regulatory system of interest without needing to know the internal design of GOMER. The software and a manual for its use are available from the GOMER web site [36].

## Additional data files

The following additional data are available with the online version of this paper. Additional data file 1 is a PDF file providing supplementary methods. Additional data file 2 is a table of the Fkh1p binding probability matrix. Additional data file 3 is a table of the Mcm1p binding probability matrix. Additional data file 4 is a table of the Sum1p binding probability matrix. Additional data file 5 is a table of the Ndt80p binding probability matrix. Additional data file 6 is a table of the Rap1p binding probability matrix. Additional data file 7 is a table listing the *CLB2 *cluster (Fkh/Mcm1 regulated genes). Additional data file 8 is a table listing open reading frames regulated by Sum1p (derepressed in a *Sum1* knockout). Additional data file 9 is a table listing listing open reading frames regulated by Ndt80p (induced by Ndt80p overexpression). Additional data file 10 is a table listing open reading frames regulated by both Sum1p and Ndt80p (intersection of Sum1p regulated ORFs and Ndt80p regulated ORFs). Additional data file 11 is a table listing open reading frames that are chromatin immunoprecipitated by Rap1p. Additional data file 12 is a table listing intergenic regions that are chromatin immunoprecipitated by Rap1p.

## Supplementary Material

Additional data file 1 Description of how PWMs, regulated gene sets, and microarray feature sequences were defined. Click here for file

Additional data file 2 This matrix was derived from binding site selection data published in [10]. Click here for file

Additional data file 3 This matrix was derived from binding site selection data published in [38]. Click here for file

Additional data file 4 This matrix was derived from *in vitro *and *in vivo *binding experiments published in [12]. Click here for file

Additional data file 5 This matrix was derived from *in vitro *and *in vivo *binding experiments published in [12]. Click here for file

Additional data file 6 This matrix was derived from a computationally defined matrix published in [13]. Click here for file

Additional data file 7 This list of *CLB2 *cluster genes was determined by expression microarray experiments published in [7]. Click here for file

Additional data file 8 This list of Sum1 regulated genes was derived from genes identified as Sum1 regulated by expression microarray experiments published in [12]. Click here for file

Additional data file 9 This list of Ndt80 regulated genes was derived from expression microarray experiments published in [5]. Click here for file

Additional data file 10 This list of Sum1 and Ndt80 regulated genes was derived from data on Sum1 regulated and Ndt80 regulated genes published in [12]. Click here for file

Additional data file 11 This list of Rap1p bound ORF sequences is derived from chromatin immunoprecipitation experiments published in [13]. Click here for file

Additional data file 12 This list of Rap1p bound intergenic sequences is derived from chromatin immunoprecipitation experiments published in [13]. Click here for file
