# Supplementary material for: Explicit equilibrium modeling of transcription-factor binding and gene regulation
Source: Genome Biol. 2005 Sep 30;6(10):R87. doi: 10.1186/gb-2005-6-10-r87 (PMC1257470; doi:10.1186/gb-2005-6-10-r87)
Supplement: Additional data file 1 — Description of how PWMs, regulated gene sets, and microarray feature sequences were defined. [file gb-2005-6-10-r87-S1.pdf]

SUPPLEMENTARY METHODS

Probability matrices for Fkh1, Mcm1Sum1, Ndt80, and Rap1

The specificity for Fkh1p (Additional File Table 1) was derived from the binding site selection data in [10]. Fkh1p binding specificity was used as a surrogate for Fkh2p, because there is no detailed binding information for Fkh2p. However existing data demonstrate that these two transcription factors have similar in vitro binding specificities [11], so we believe that substituting the Fkh1p PM for Fkh2p binding specificity is a reasonable approximation. The binding specificity matrix for Mcm1p (Additional File Table 2) was derived from the binding site selection data in [38]. In generating the Fkh1 and Mcm1 matrices, we have assumed that positions having base counts of zero are a consequence of incomplete sampling. To account for this, a single pseudocount was added to every base count in every position. The base counts in each position were scaled to base probabilities such that the probabilities in a position sum to unity.

The Sum1 and Ndt80 PMs (Additional File Tables 3 and 4) were derived from the in vitro binding data of [12] and correspond to positions 3-13 of the MSE consensus sequence indicated in that paper. Positions outside this region were not used because the data are incomplete and because there appears to be little specificity information outside this region. In this dataset, some substitutions within the binding site were not tested in vitro, but were tested in vivo. For these positions we interpolated binding by fitting a logarithmic function to the relationship between in vivo and in vitro values. The percent bound values for each base in each position were scaled to base probabilities such that the probabilities in a position sum to unity.

The Rap1 PM (Additional File Table 5) was derived from the "Top-scoring Motif Matrix" of the Bioprospector predicted binding specificities of Web Table E in [13]. The values for each base in each position were scaled to base probabilities such that the probabilities in a position sum to unity.

Mcm1 and Fkh2 Regulation

The regulated gene set used for the analysis of Mcm1 and Fkh2 (Additional File Table 6) consists of the genes of the CLB2 cluster, as reported in Figure 4C of [7].

The Sum1 and Ndt80 Regulatory System

The Sum1-regulated genes are derived from those genes found to be upregulated in a *sum1Δ* strain as compared to a wild-type strain [12]. Ignoring genes that are labeled "Dubious" in the genome annotation, there are 49 Sum1-regulated genes (Additional File Table 7). The Ndt80-regulated set was determined from expression microarray experiments comparing cells expressing high levels of Ndt80p with wild-type cells [5]. Differential gene expression ratios were calculated using

$$\left(\frac{(\text{gal-ndt80 green}) - (\text{gal-ndt80 green bkg})}{(\text{gal-ndt80 red}) - (\text{gal-ndt80 red bkg})} \right)$$

where the terms refer to column names in data that can be obtained at [5]. Genes found to have at least three-fold higher expression under high Ndt80p conditions were considered to be Ndt80-regulated. Ignoring features that are labeled "Pseudogene" or "Dubious" in the annotation, and features that no longer exist in the genome annotation the Ndt80-regulated set consists of 181 genes (Additional File Table 8). The intersection of the Ndt80-regulated and Sum1-regulated gene sets consists of 30 genes (Additional File Table 9). When determining the ROC AUC values for each of these three sets, we excluded the genes in the other two sets from the analysis. For example, when determining the ROC AUC for the Ndt80 set, all of the genes in the Sum1 set and the intersection set were ignored (they were considered as neither regulated nor unregulated).

We calculated GOMER scores for Ndt80 binding alone, and for Ndt80 competed by Sum1. The competition between Ndt80p and Sum1p was analyzed at a range of Sum1 free concentration ratios, using a square competition weight function, which gave a weight of one to all competing sites that overlapped the primary binding site at all, or were no more than one base away.

Microarray Sequence Coordinates

The Rap1 ChIP data of [13] that was used in this paper was obtained from whole genome spotted microarrays. The DNA spotted on these arrays were generated by PCR amplification of yeast genomic DNA using the "Yeast GenePairs® Primers" and "Yeast Intergenic Region Primers" from Research Genetics. We obtained the primer sequences from Invitrogen Technical Support. However the supplied base coordinates for the sequences that are expected to be amplified are almost all incorrect for recent versions of the *S. cerevisiae* genome; additionally, based on recent versions of the genome, many primer pairs are expected to fail to amplify any product. Therefore, we determined the base coordinates of expected PCR products corresponding to the version of the genome we used. To do this, the unique portions of each primer pair were extracted (i.e. the "universal" sequences were ignored), and were used to search the genome for predicted PCR products, using the **primersearch** program [39]; no mismatches to the primer sequences were allowed. The PCR product was considered to be well determined (and therefore successfully amplified and spotted on the microarray) if **primersearch** predicted exactly one PCR product that was 7000bp or shorter. Primer pairs that produced multiple predicted PCR products were not considered acceptable unless products were nested, i.e. every product was a subsequence of all products larger than itself. In this case, the innermost (smallest) product was assumed to be the only amplified sequence. Multiple predicted products were also considered acceptable in the special case of the silent mating type loci as these are known to represent perfect duplications. A PCR product was considered to be a gene if its primers occurred in the "Yeast GenePairs® Primers" set, and considered to be intergenic if in the

“Yeast Intergenic Region Primers” set. There are a small number of ORFs (forty-two) for which primer pairs exist in both the “Yeast GenePairs® Primers” set and in the “Yeast Intergenic Region Primers” set. For most of these ORFs (thirty-six) the primers from both sets find acceptable amplimers. In these cases, the coordinates for the “Yeast GenePairs® Primers” amplimer are used, and these features are considered to be ORFs. For two of the ORFs for which there are primer pairs given in both lists, only the “Yeast GenePairs® Primers” find acceptable amplimers the coordinates for these amplimer are used, and these features are considered to be ORFs. Finally, for two of the ORFs for which there are primer pairs in both lists, only the primers from the “Yeast Intergenic Region Primers” set find acceptable amplimers. For these two ORFs, coordinates derived for the primers listed in the “Yeast Intergenic Region Primers” are used to define the sequence on the array, and these are considered to be intergenic features. The primer coordinates used and tools for extracting them are available at the GOMER web site [36].
